# Now You Feel *both*: Galvanic Vestibular Stimulation Induces Lasting Improvements in the Rehabilitation of Chronic Tactile Extinction

**DOI:** 10.3389/fnhum.2013.00090

**Published:** 2013-03-20

**Authors:** Lena Schmidt, Kathrin S. Utz, Lena Depper, Michaela Adams, Anna-Katharina Schaadt, Stefan Reinhart, Georg Kerkhoff

**Affiliations:** ^1^Clinical Neuropsychology Unit and Outpatient Service, Saarland UniversitySaarbruecken, Germany; ^2^International Research Training Group 1457 “Adaptive Minds,”Saarbruecken, Germany; ^3^Department of Neurology, University of Erlangen-NurembergErlangen, Germany

**Keywords:** body, extinction, vestibular, touch, brain recovery, awareness, rehabilitation

## Abstract

Tactile extinction is frequent, debilitating, and often persistent after brain damage. Currently, there is no treatment available for this disorder. In two previous case studies we showed an influence of galvanic vestibular stimulation (GVS) on tactile extinction. Here, we evaluated in further patients the immediate and lasting effects of GVS on tactile extinction. GVS is known to induce polarity-specific changes in cerebral excitability in the vestibular cortices and adjacent cortical areas. Tactile extinction was examined with the Quality Extinction Test (QET) where subjects have to discriminate six different tactile fabrics in bilateral, double simultaneous stimulations on their dorsum of hands with identical or different tactile fabrics. Twelve patients with stable left-sided tactile extinction after unilateral right-hemisphere lesions were divided into two groups. The GVS group (*N* = 6) performed the QET under six different experimental conditions (two Baselines, Sham-GVS, left-cathodal/right-anodal GVS, right-cathodal/left-anodal GVS, and a Follow-up test). The second group of patients with left-sided extinction (*N* = 6) performed the QET six times repetitively, but without receiving GVS (control group). Both right-cathodal/left-anodal as well as left-cathodal/right-anodal GVS (mean: 0.7 mA) improved tactile identification of identical and different stimuli in the experimental group. These results show a generic effect of GVS on tactile extinction, but not in a polarity-specific way. These observed effects persisted at follow-up. Sham-GVS had no significant effect on extinction. In the control group, no significant improvements were seen in the QET after the six measurements of the QET, thus ruling out test repetition effects. In conclusion, GVS improved bodily awareness permanently for the contralesional body side in patients with tactile extinction and thus offers a novel treatment option for these patients.

## Introduction

In daily life touch is important in many situations, i.e., when we grasp objects, manipulate them, or identify them, e.g., when retrieving a key from our pocket. Brain lesions, due to stroke, head trauma, or other causes impair a variety of somatosensory abilities dramatically in more than 50% of patients (Van Stralen et al., 2011). Among these impairments, tactile or somatosensory extinction is a frequent disorder (Kerkhoff et al., 2011). Extinction of sensory stimuli – in whatever modality – is defined as the inability to process or attend to the more contralesionally located stimulus when two stimuli are simultaneously presented. By definition, the processing of a single stimulus should only be marginally impaired, thereby ruling out gross elementary sensory deficits (i.e., hemianopia, hemianesthesia, unilateral hearing loss). Extinction may occur in the visual (Conci et al., 2009), auditory (Deouell and Soroker, 2000), olfactory (Eskenazi et al., 1983), or tactile modality (Berti et al., 1999; Maravita et al., 2003). Tactile extinction is frequently found after unilateral, mostly right-sided brain lesions (70%, Schwartz et al., 1977, 1979; Heldmann et al., 2000), is a negative predictor for the patient’s outcome (Rose et al., 1994), and often persists for years after lesion (Heldmann et al., 2000). Causative lesions are found in the frontal, parietal or temporal cortex (Schwartz et al., 1977; Deouell and Soroker, 2000), and the basal ganglia (Vallar et al., 1994). In addition, anterior callosal lesions may disrupt the processing of the left hand tactile stimulus (Schwartz et al., 1979), which may explain the more frequent occurrence of tactile extinction on the left body side than on the right (Schwartz et al., 1979). Moreover, tactile extinction does not only occur when the patient has to *detect* tactile stimulation (Bender, 1952), but also appears when he/she has to *discriminate* different tactile surfaces (Schwartz et al., 1977), and even occurs when a patient simultaneously explores two common household objects actively by touch (Berti et al., 1999). Tactile extinction is modulated by stimulus properties (i.e., additional sensory stimulation of the hand) *and* response factors (verbal vs. non-verbal output; cf. Vaishnavi et al., 2000). The latter indicates that interference between both stimuli can even occur at a post-perceptual level, probably close to the language system.

Two main explanations of extinction have been proposed: sensory (Bender, 1952) and attentional theories (Vallar et al., 1994). While the prior explains extinction as the result of a weakened sensory integration process, the latter holds that elementary sensory abilities may be completely intact, and yet extinction occurs. In favor of the latter account, several studies have shown that early sensory or pre-attentive processes are often reasonably intact in patients with visual extinction (Conci et al., 2009). Various stimulation maneuvers such as caloric vestibular stimulation (Vallar et al., 1993), optokinetic stimulation (Nico, 1999), repetitive peripheral magnetic stimulation (RPMS) (Heldmann et al., 2000), visuomotor prism adaptation (Maravita et al., 2003), or positioning of the “extinguishing” limb in the ipsilesional hemispace (Aglioti et al., 1999; Sambo et al., 2012) significantly modulate tactile extinction. This accords with proposals that somatosensory deficits in right-hemisphere patients may relate, at least partially, to neglect (Vallar, 1997), which can be significantly modulated by sensory stimulation maneuvers (Kerkhoff, 2003). Yet, few studies have so far evaluated to which degree tactile extinction can be *permanently* cured with such methods. A remarkable case study (Dijkerman et al., 2004) reported a long-lasting (for at least 1–3 weeks), beneficial effect of only *two* sessions of prism adaptation on somatosensory functions (pressure sensitivity and proprioception), indicating a considerable capability for the treatment of these disorders. Other sensory stimulation techniques might induce similar beneficial effects on somatosensory deficits after stroke, thus offering a potential treatment choice beyond the classic therapies already available for a longer time (cf. Carey, 1995; Carey and Matyas, 2005).

One such technique is *galvanic* vestibular stimulation (GVS). GVS is a non-invasive vestibular stimulation that is, unlike *caloric* vestibular stimulation, easier to use, lacking adverse side effects (with currents <1.5 mA) and therefore appears more appropriate for *repetitive* treatment without habituation effects (Utz et al., 2010, 2011b). Practically, weak direct currents (DCs) are delivered via two electrodes of different polarity (anode and cathode) attached to the two mastoids behind the ears. On the neural level, GVS induces polarization effects in the vestibular nerves, leading to an activation of the semicircular canals, otolith organs, and the adjacent vestibular nerves (Fitzpatrick and Day, 2004). Cortical activation is seen in the posterior insula and the temporo-parietal region in healthy subjects during GVS. Further activation was found in the middle and superior temporal gyrus, the putamen, the anterior cingulate gyrus, and thalamus (Lobel et al., 1998; Bense et al., 2001). Interestingly, *bilateral* activations of vestibular cortices are obtained by applying left-cathodal/right-anodal GVS (further termed L-GVS), whereas *unilateral*, right-hemispheric activations are induced by right-cathodal/left-anodal GVS (further termed R-GVS) (Dieterich et al., 2003; Fink et al., 2003).

Only a few studies have so far evaluated the potency of GVS in patients with neglect, extinction, and related spatial disorders. Rorsman et al. (1999) showed a transient reduction of visual neglect symptoms in patients with neglect (i.e., line cancelation) during R-GVS. A recent case study found a significant improvement in visuo-constructive deficits (copy of Rey-figure) during GVS (Wilkinson et al., 2010). Recently, we have already been successful in modulating neglect with GVS: one 20 min session of R-GVS temporarily reduced the ipsilesional bias in line bisection (Utz et al., 2011a), whereas 20 min of L-GVS normalized the profound deficits in left arm position sense in patients with left neglect (Schmidt et al., 2013).

As outlined above, GVS can modulate the thalamocortical network of the brain in a polarity-specific way, either by activation (anodal stimulation) or de-activation (cathodal stimulation) (Utz et al., 2010). As tactile extinction is viewed by some theories (Schwartz et al., 1979) as resulting from an imbalance of somatosensory inputs received simultaneously from both hands we hypothesized that GVS may re-balance this disturbed weighting via activations of certain brain areas involved in tactile extinction or inhibition of mirror-symmetric areas in the intact hemisphere. In two recent case studies we could show a lasting influence of a few sessions of GVS on tactile extinction (Kerkhoff et al., 2011), thus serving as an initial proof-of-principle test of the therapeutic efficacy of GVS.

**Figure 1:**
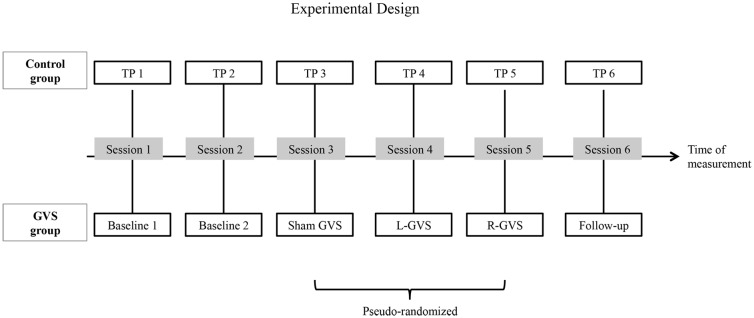
**A schematic overview of the experimental design: the galvanic vestibular stimulation (GVS) conditions performed with the six experimental patients with extinction and the different time-points of measurement (TP) performed with the six control patients with extinction, respectively in six different sessions**. Abbreviations: L-GVS, left-cathodal/right-anodal GVS; R-GVS, right-cathodal/left-anodal GVS; Sham, Sham stimulation with GVS but without the application of current; Follow-up, mean follow-up 2.8 months (84 days) after GVS.

Furthermore, promising effects of GVS in the modulation and/or treatment of other symptoms associated with neglect syndrome (Kerkhoff and Schenk, 2012) initiated to study the effects of GVS on tactile extinction in a larger sample, including a non-treated control group showing the same disorder as the treated, experimental group. From available literature on GVS we expected a *transient* reduction of left-sided (left hand) extinction errors under GVS, but no specific effect on right-sided (right-hand) errors induced by GVS. Regarding polarity we had no directional hypothesis as some of the few available studies on GVS showed improvements during L-GVS, whereas others showed improvements during R-GVS (as mentioned above). In the present study we therefore explored the effects of GVS on tactile extinction in two comparable samples of patients with right-sided stroke (experimental group: *N* = 6, control group: *N* = 6), all showing left-sided tactile extinction. Apart from *online*-stimulation effects (*during* GVS) we were particularly interested in the *after*-effects of GVS and potential enduring treatment effects on tactile extinction in the experimental group. In the control group, the influence of retesting was analyzed by testing the patients six times in an identical study protocol, but without GVS (see below, Figure 1).

## Materials and Methods

### Participants

A total of 12 patients with right-hemisphere stroke and left-sided tactile extinction as determined in the Quality Extinction Test (QET; see below) were included in the study. Six patients served as the experimental group (four males, GVS group) and received different protocols of GVS, while the other six patients served as the control group (three males, control group) which was retested six times with the QET in identical schedule to rule out test repetition and other unspecific effects (Table 1). Allocation of patients into the two patient groups was done in the following way: first, six experimental patients with extinction were treated with GVS as described below; second, six control patients with extinction were recruited in order to match the sample of experimental patients in demographic and clinical variables and extinction severity. Time intervals between the six different sessions were identical between the two patient groups. They did not differ with respect to age [*T*(10) = 1.526, *p* = 0.236], sex [χ^2^(*df* = 1) = 3.43, *p* = 0.558], or time since lesion [*T*(10) = 1.541, *p* = 0.154]. They did not differ in their Baseline performances in the QET, neither for their left or right-hand nor for different or identical materials (all *p*s > 0.05). All subjects except one were right-handed according to the Edinburgh handedness questionnaire (Salmaso and Longoni, 1985) and had no history of psychiatric disorders or dementia. A visual neglect screening including digit cancelation (cancel all digits “5” out of 200 single digits on a 21 cm × 29.7 cm large white paper, 10 targets per hemispace), horizontal line bisection of a 20 cm × 0.5 cm long black line and text reading of a 180 word reading text were conducted in all patients (details of these tests in Schmidt et al., 2013). All investigations were performed in accordance with the Declaration of Helsinki II and all participants gave their informed written consent before examination. A positive, written ethical approval by the local medical ethical committee (Ärztekammer Saarland) was available for the use of subliminal GVS in brain-damaged patients. No patient was enrolled in any other neuropsychological treatment (attention, neglect) or motor therapy (physiotherapy, occupational therapy) during the course of the study.

**Table 1:** **Clinical and demographic data of 12 patients with left-sided tactile extinction due to a right-hemisphere brain lesion**.

Patient	Group	Age, sex	Handedness	Etiology	Lesion, Lesion age (months)	Motor deficits	Visual field	Digit cancelation omissions L/R max. (10/10)	Line bisection (20 cm, deviation in mm)	Neglect dyslexia	Visual neglect	Tactile extinction
1-LA	GVS	70, Male	Right-hander	ICB	Right fronto-parietal, 60.3	Left hemiparesis	Normal	0/0	−3	No	Yes	Yes
2-RE	GVS	45, Female	Right-hander	ICB	Right frontal, right temporal, 71.2	Left hemiparesis	Left hemianopia, 10°	1/1	+2	No	Yes	Yes
3-KA	GVS	66, Male	Right-hander	ICB	Right parietal, 6	Left hemiparesis	Normal	4/1	+13	Yes	Yes	Yes
4-NI	GVS	51, Female	Right-hander	MCI	Right fronto-parietal, 6	Left hemiparesis	Normal	5/2	−10	No	Yes	Yes
5-SC	GVS	72, Male	Right-hander	PCI	Right occipital, right thalamus, 25	Normal	Left hemianopia	4/1	−12	Yes	Yes	Yes
6-KL	GVS	47, Male	Left hander	Thalamus infarction	Right pulvinar, 2.3	Normal	Left upper quadranopia	2/1	−7	Yes	No	Yes
Mean	*N* = 6	58.3 (SD = 12.4)			28.5 (SD = 30.2)			3/1	−2.8			
7-ME	Control	59, Male	Right-hander	ICB	Right fronto-parietal, 8	Left hemiparesis	Normal	2/2	−4	Yes	No	Yes
8-TA	Control	47, Female	Right-hander	ICB	Right basal ganglia, 12	Left hemiparesis	Normal	2/1	−2	No	Yes	Yes
9-CR	Control	68, Male	Right-hander	PCI	Right thalamus and right occipital, 5	Normal	Left hemianopia	5/2	+11	Yes	Yes	Yes
10-WI	Control	45, Male	Right-hander	MCI	Right parietal, 15	Left hemiparesis	Left lower quadranopia	4/2	+9	Yes	Yes	Yes
11-TU	Control	47, Female	Right-hander	ICB	Right parietal, 5	Left hemiparesis	Left lower quadranopia	4/2	+10	Yes	Yes	Yes
12-HA	Control	25, Female	Right-hander	ICB	Right basal ganglia, 11	Normal	Normal	1/1	+3	No	No	Yes
Mean	*N* = 6	48.5 (SD = 14.6)			9.3 (SD = 4.0)			3/2	+4.5			

*ICB, intracerebral bleeding; PCI, posterior cerebral artery infarction; MCI, middle cerebral artery infarction. Visual neglect: diagnosis based on conventional tests of digit cancelation, line bisection, and reading (for details, see Schmidt et al., 2013); tactile extinction: as tested by tactile stimulation of the patient’s dorsum of hands by light touch*.

### Quality extinction test

The QET (Schwartz et al., 1977) is a sensitive tactile extinction test that requires the subject to identify and name six different tactile surfaces first in unilateral trials on the left and right dorsum of hands and then in double simultaneous stimulation (DSS) trials with the same materials. Previous studies with the QET have shown that patients with right frontal or right parietal lesions consistently show marked left-sided tactile extinction in those trials with *bilateral* different stimuli while showing normal performance in unilateral target presentations (Schwartz et al., 1977, 1979). Subsequent studies with the QET provided evidence that tactile extinction is modulated by somatosensory input delivered via RPMS of the left forearm (Heldmann et al., 2000; Kerkhoff et al., 2001). Moreover, we found that apart from those bilateral trials with *different* fabrics (e.g., left hand: sandpaper, right-hand: silk) those bilateral stimulations using *identical* fabrics (e.g., both hands: silk) also made a useful diagnostic contribution, although they appeared to be easier to solve (cf. Kerkhoff et al., 2011).

The present version of the QET includes six different materials varying in tactile quality (soft sandpaper, silk, fleece, plastic, jute, and rubber gum) that were attached singly to wooden boards (size: 15 cm × 10 cm). Patients placed their hands with palms down and beside each other (hence in the normal “anatomical” position) on the table in front of the experimenter. During all testing sessions patients were blindfolded and wore a closed head-phone in order to prevent visual and auditory cues during the tactile stimulation procedure. Patients were instructed to identify and name the six different tactile materials used throughout the test. To this purpose, single boards were moved slowly by the experimenter with a speed of 2 cm/s from proximal to distal across the dorsum of either the left or right-hand. Each material was presented three times in this way and the patients had to report the material verbally. Twelve unilateral trials were run for each hand separately per patient, for every testing session. After these unilateral trials, which served to assess unilateral tactile performance, bilateral stimulation trials were performed. Here, two boards were presented simultaneously, one to each hand, and the patient had to name the material(s) he/she recognized on each hand. A total of 36 bilateral trials were performed in each complete test: 18 trials with different and 18 trials with identical materials delivered to both hands. Unilateral trials were not repeated during the experimental sessions as normal or near-to-normal unilateral performance had been established already in the two Baseline sessions before GVS. Moreover, the unilateral trials were of no particular interest in this study after normal unilateral performance had been established in all patients. Patients were unaware of the fact that one half of the trials were performed with identical and the other half with different tactile materials as both were intermingled within every session, but they were instructed that materials can be identical or different for both hands. If patients could not identify correctly one or both of the materials in a trial with bilateral stimulation, an extinction response was scored for the corresponding side. Thereafter, the next bilateral stimulation trial was performed. No attempt was made to force the patients to guess in case they were unable to verbally identify the material. The patients were not forced to guess whether the two stimuli were same or different in case of missing verbal response for one side. No time constraints were imposed and no feedback was given during testing. The percentage and raw score of left- and right-sided extinction during DSS with *different* tactile stimuli (based on 18 trials) as well as during DSS with *identical* tactile stimuli (based on the other 18 trials) were computed for every session. Note that the QET – in contrast to conventional tactile extinction procedures using light touches of the patient’s hands – requires *discrimination* of six different tactile materials and finally their verbal identification. Therefore, a higher degree of error rates may be found, including some ipsilesional errors as well (Heldmann et al., 2000). Chance level, i.e., when the patient is guessing, is 16.6% in this task.

### Galvanic vestibular stimulation

Bipolar GVS was delivered by a constant DC stimulator (9-V battery, Type: ED 2011, producer: DKI GmbH, DE-01277 Dresden, Germany). The tap water-soaked sponge-covered electrodes (60 mm × 40 mm) were fastened on the skin over each mastoid (binaural stimulation) in order to activate the vestibular system. For L-GVS the cathode was placed on the left mastoid and the anode on the right, whereas for R-GVS this electrode setup was reversed. In the Sham-GVS condition, the two electrodes were positioned as in the L-GVS condition, except that no electric current was applied in order to rule out potential placebo-stimulation effects. We stimulated below the sensation threshold (subliminal) so that the subject was not aware of any electrical stimulation in any experimental or sham condition (Utz et al., 2010). As there is evidence that even subtle attentional cues can modulate neglect and extinction (Riddoch and Humphreys, 1983), we employed this subliminal stimulation as it elegantly circumvents potential attentional cueing effects that might occur with *supra-threshold* stimulation. A switch on the stimulation device delivered current at an individually adjusted level to the patients. The individual threshold was determined by slowly increasing current intensity in steps of 0.1 mA until the patient indicated a tingling. Current was then reduced until the patient indicated that the sensation had disappeared. This procedure was repeated a second time and the mean of both threshold values was defined as the individual threshold. Individual thresholds of each patient were determined at the beginning of both stimulation sessions (L-GVS, R-GVS) in order to exclude supra-threshold stimulation caused by reduced thresholds for GVS (see results, below). Finally, in all conditions, the GVS stimulator was never visible for the patients.

### Experimental design

Patients in both groups participated in six different sessions (see Figure 1 for an outline of the design). In the control group, six investigations were performed with the QET at six different time-points of measurement (TP, 1–6) without GVS stimulation. In contrast, patients in the experimental (GVS) group performed two Baseline sessions without GVS. In session three to five, they performed the QET again while receiving either L-GVS, R-GVS, or Sham-GVS, respectively in a pseudo-randomized sequence to control for order effects. Subjects were blind to the type of stimulation received. A follow-up was conducted 84 days [=2.8 months (mean); range: 35–147 days] after the fifth testing session in all subjects (hence after the last GVS session in the experimental group and after TP5 in the control group). A 2-day interval (min. 48 h) was established between sessions to avoid carry-over effects. Importantly, the timing of testing sessions was identical in both samples (see Figure 1).

### Statistics

All analyses were carried out using SPSS, version 19. First, we calculated extinction errors (in %) in the QET, separately for the 18 different and 18 identical bilateral trials, for each hand and each group. Repeated-measures analyses of variance (ANOVAs) with the between factor “group” (GVS group, control group) and the within factor “GVS condition/TP” (Baseline 1/TP1, Baseline 2/TP2, Sham/TP3, L-GVS/TP4, R-GVS/TP5, Follow-up/TP6) were carried out separately for the right and left hand and for different and identical stimuli. Subsequent comparisons [ANOVAs and Bonferroni-adjusted *t*-tests for multiple comparisons (Holm, 1979)] were computed for a more specific examination of significant results. The alpha-level was set at *p* = 0.05, two-tailed for all analyses.

## Results

### Unilateral trials

In the 24 unilateral trials (12 unilateral trials per measurement × 2 measurements) each of the 12 patients scored >95% correct for the left hand and >98% for the right-hand in the QET, thus showing normal or close-to-normal unilateral tactile identifications for both hands.

### Analysis of baseline 1 vs. baseline 2

Analyses of variances with the between factor “group” (GVS group, control group) and the within factor “TP” (Baseline 1, Baseline 2), separately for different and identical materials and for each hand, revealed no significant effects of these factors, suggesting that there were no differences between the two first time-points of assessment (Baseline 1, 2) in the two groups (largest *F* = 3.88, smallest *p* = 0.077).

### Individual threshold values and side effects of GVS

The mean current level at GVS threshold in the GVS group was 0.7 mA (range: 0.5–0.8 mA). This averaged threshold did not differ significantly between L-GVS (TP4) and R-GVS (TP5) condition (*Z* = −1.0, *p* = 0.317). A 34-items-questionnaire regarding possible side effects of GVS stimulation, which included items about fatigue, dizziness, vision and sleep disturbances, concentration difficulties, pain, skin disturbances, burning sensations, etc. (cf. Utz et al., 2011b) was read by the examiner to all six patients after every real and sham stimulation. No adverse effects were reported by any of the six experimental patients during or after GVS, except a slight tingling at the beginning of stimulation in the course of the individual threshold determination that was not negatively evaluated, but rather indicated that real current was delivered during GVS stimulation. Table 2 summarizes the individual and mean threshold values as well as side effects in the experimental group.

**Table 2:** **Individual and mean threshold values (milliAmpere, mA) for subliminal GVS conditions for patients in the GVS group and mean number of side effects (%) according to the 34-items-questionnaire, averaged over the GVS group and separately for each GVS condition**.

Patient	L-GVS	R-GVS
**THRESHOLD VALUES (mA)**		
1-LA	0.5	0.6
2-RE	0.5	0.5
3-KA	0.8	0.8
4-NI	0.7	0.7
5-SC	0.8	0.8
6-KL	0.6	0.6
Mean	0.7	0.7
Side effects (%)	0	0

*L-GVS, left-cathodal/right-anodal GVS; R-GVS, right-cathodal/left-anodal GVS*.

### Bilateral different tactile stimulation

#### Right-hand

**Figure 2:**
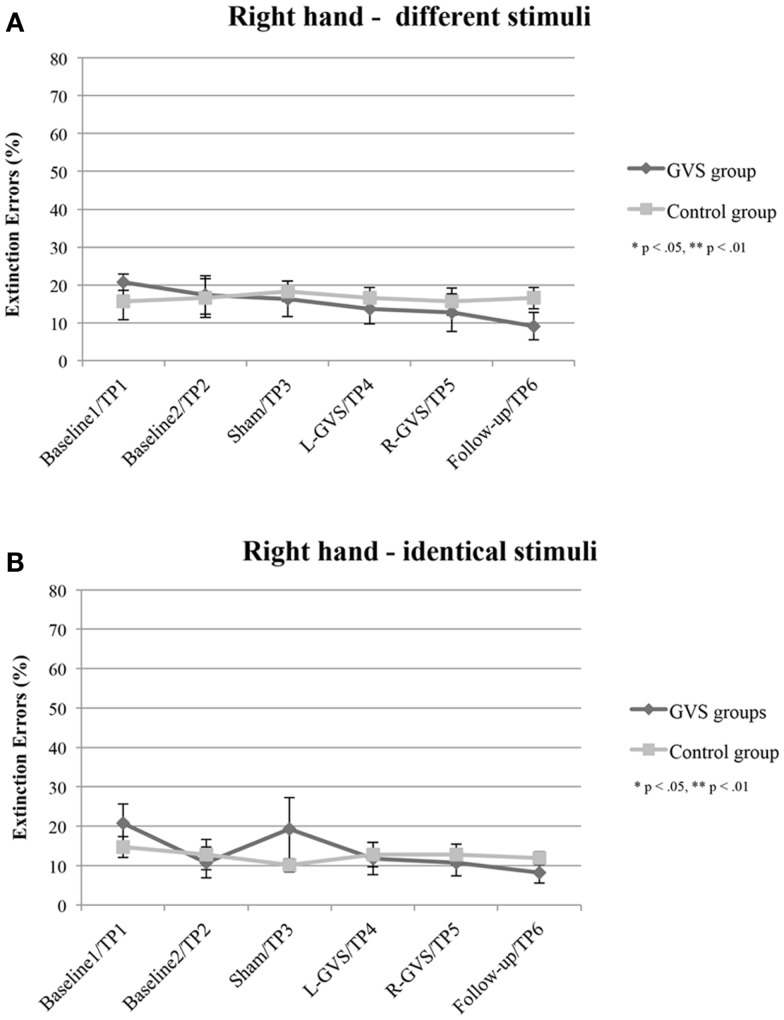
**Mean (±standard error of the mean) extinction errors (%) for the right-hand in the Quality Extinction Test (QET) of the GVS group (*N* = 6) and control group (*N* = 6) across the six measurement sessions for application of *different* tactile stimuli (A) and of *identical* tactile stimuli (B)**. Note that apart from moderate variations in error rates no significant improvement was observed in the control group due to retesting in six subsequent sessions. Abbreviations: L-GVS, left-cathodal/right-anodal GVS; R-GVS, right-cathodal/left-anodal GVS; Sham, Sham stimulation with GVS but without the application of current; Follow-up, follow-up 2.8 months after GVS.

The analysis of extinction errors during bilateral stimulation with *different* tactile stimuli of the right-hand did not show statistically significant main effects of GVS condition/TP [*F*(5,50) = 1.01, *p* = 0.424, η^2^ = 0.091] or group [*F*(1,10) = 0.14, *p* = 0.718, η^2^ = 0.014] or a significant GVS condition/TP × group interaction [*F*(5,50) = 1.03, *p* = 0.407, η^2^ = 0.094] (see Figure 2A).

#### Left hand

**Figure 3:**
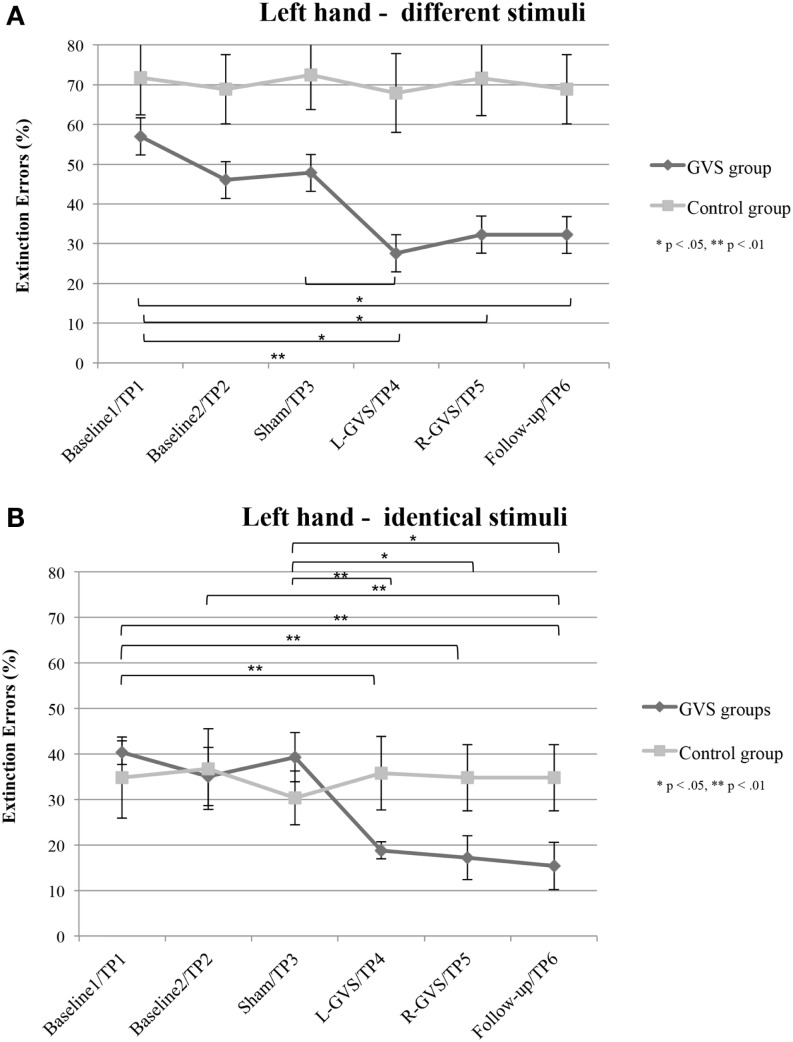
**Mean (±standard error of the mean) extinction errors (%) for the left hand in the Quality Extinction Test (QET) of the GVS group (*N* = 6) and control group (*N* = 6) across the six measurement sessions for application of *different* tactile stimuli (A) and of *identical* tactile stimuli (B)**. Note that apart from moderate variations in error rates no significant improvement was observed in the control group due to retesting in six subsequent sessions. Abbreviations: see legend of Figure 2.

In contrast, the analysis of left hand extinction scores during bilateral stimulation with *different* tactile materials yielded a significant main effect of GVS condition/TP [*F*(5,50) = 5.99, *p* = 0.003, η^2^ = 0.375], of group [*F*(1,10) = 8.76, *p* = 0.014, η^2^ = 0.467] as well as a significant interaction between these two factors [*F*(5,50) = 4.17, *p* = 0.015, η^2^ = 0.294]. Subsequent ANOVAs were carried out separately for the two patient groups with the factor GVS condition/TP to examine simple main effects. They yielded a significant main effect of GVS condition/TP only for the GVS group [*F*(5,25) = 5.57, *p* = 0.001, η^2^ = 0.527] but not for the control group [*F*(5,125) = 1.23, *p* = 0.326, η^2^ = 0.197]. Subsequent *t*-tests analyzing the extinction errors differences between different GVS conditions/TP in the GVS group showed significant improvements in left-sided extinction in the L-GVS [*T*(5) = 7.53, *p* = 0.001], the R-GVS [*T*(5) = 3.43, *p* = 0.019], and the Follow-up [*T*(5) = 3.12, *p* = 0.024] condition as compared to Baseline 1. Likewise, patients in the GVS group showed a less severe extinction in the L-GVS as compared to the Sham condition (*T*(5) = 2.91, *p* = 0.034). The remaining comparisons did not show any significant differences between any of the conditions (largest *T* = 2.28, smallest *p* = 0.071) (see Figure 3A). There were no differences between extinction errors in the L-GVS and R-GVS condition for the left hand in different materials [*T*(5) = −0.63, *p* = 0.558]. Table 3 (below) summarizes the results of the paired comparisons in the GVS group for the left hand, for easier orientation.

**Table 3:** **Summary of paired comparisons between the different GVS conditions for the left hand of the GVS group, separately for different and identical tactile stimuli**.

	Baseline 1	Baseline 2	Sham	L-GVS	R-GVS	Follow-up
**DIFFERENT STIMULI**						
Baseline 1	–	n.s.	n.s.	**	*	*
Baseline 2	–	–	n.s.	n.s.	n.s.	n.s.
Sham	–	–	–	*	n.s.	n.s.
L-GVS	–	–	–	–	n.s.	n.s.
R-GVS	–	–	–	–	–	n.s.
Follow-up	–	–	–	–	–	–
**IDENTICAL STIMULI**						
Baseline 1	–	n.s.	n.s.	**	**	**
Baseline 2	–	–	n.s.	n.s.	n.s.	n.s.
Sham	–	–	–	**	*	*
L-GVS	–	–	–	–	n.s.	n.s.
R-GVS	–	–	–	–	–	n.s.
Follow-up	–	–	–	–	–	–

*L-GVS, left-cathodal/right-anodal GVS; R-GVS, right-cathodal/left-anodal GVS; Sham, Sham stimulation with GVS but without the application of current; Follow-up, follow-up 2.8 months after GVS*.

*p < 0.05, **p < 0.01.

### Bilateral identical tactile stimulation

#### Right-hand

There were no significant effects of GVS condition/TP [*F*(5,50) = 1.49, *p* = 0.211, η^2^ = 0.129], group [*F*(1,10) = 0.09, *p* = 0.770, η^2^ = 0.009], or of the interaction [*F*(5,50) = 1.39, *p* = 0.246, η^2^ = 0.122], when analyzing error scores in the *identical* stimulation condition (see Figure 2B).

#### Left hand

The analysis of variance of errors during bilateral stimulation with *identical* stimuli revealed a significant effect of GVS condition/TP [*F*(5,50) = 5.82, *p* = 0.000, η^2^ = 0.368] and of the GVS condition/TP × group interaction [*F*(5,50) = 7.64, *p* = 0.000, η^2^ = 0.433] but not of the factor group [*F*(1,10) = 0.72, *p* = 0.418, η^2^ = 0.067]. Further analyses of identical tactile stimuli scores yielded a significant main effect of GVS condition/TP only for the GVS group [*F*(5,25) = 8.33, *p* = 0.000, η^2^ = 0.625], but not for control patients [*F*(5,25) = 1.06, *p* = 0.407, η^2^ = 0.175]. Moreover, subsequent *t*-tests for left-sided extinction scores showed the following differences between GVS conditions for the GVS group: the initial Baseline 1 score was significantly higher than during L-GVS [*T*(5) = 7.39, *p* = 0.001], R-GVS [*T*(5) = 9.49, *p* = 0.000], and Follow-up [*T*(5) = 6.52, *p* = 0.001] and patients showed a significant improvement in left-sided extinction in the Follow-up condition as compared to Baseline 2 [*T*(5) = 2.63, *p* = 0.047]. Furthermore, we found a significant improvement in extinction scores under L-GVS [*T*(5) = 5.39, *p* = 0.003], R-GVS [*T*(5) = 3.11, *p* = 0.026], and in the Follow-up [*T*(5) = 3.3, *p* = 0.021] as compared to Sham condition. All other comparisons missed significance (largest *T* = 2.54, smallest *p* = 0.054) (see Figure 3B). There were no differences between extinction errors in the L-GVS and R-GVS condition for left hand in identical materials [*T*(5) = 0.38, *p* = 0.722]. Table 3 gives a summary of the paired comparisons for the left hand in the GVS group.

### Additional analyses

A closer look at data of Baseline 1 yielded that tactile extinction was significantly more severe (as shown by higher error rates in the QET) for both groups, when *different* tactile stimuli had to be discriminated on the left hand (mean: 64.3%) as compared to the condition with identical tactile stimuli [mean: 37.6%; *T*(11) = −4.52, *p* = 0.001]. No such difference was obtained for the right-hand [mean error rate for different vs. identical stimuli: 18.1 vs. 17.8%, *T*(11) = 0.106, *p* = 0.918].

**Figure 4:**
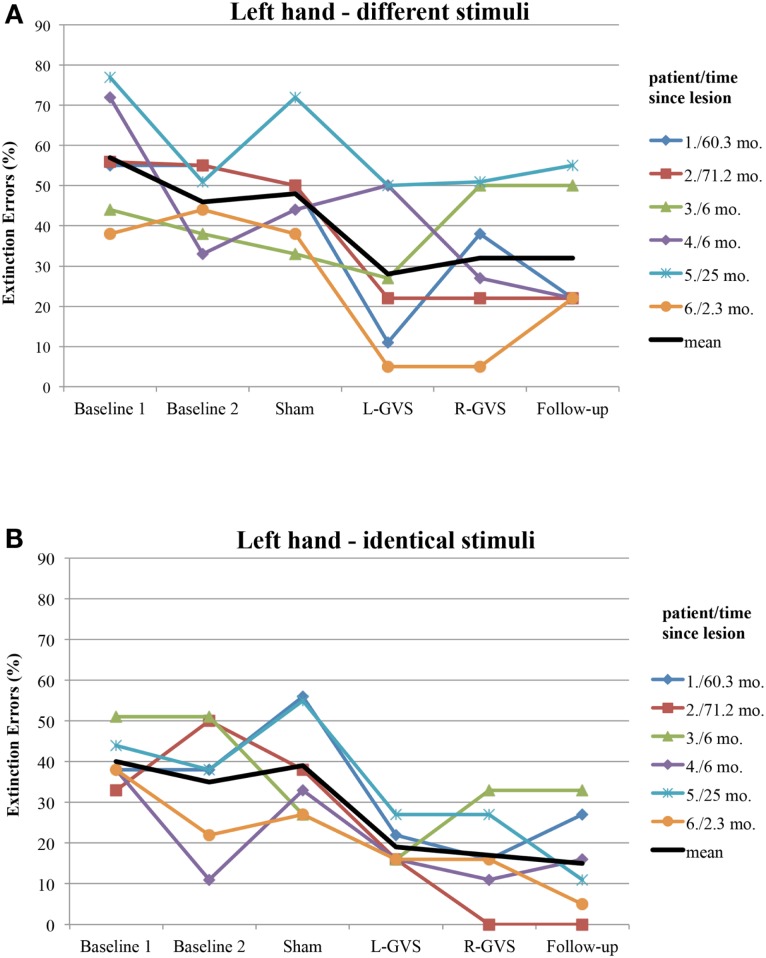
**Individual extinction errors (in degrees, averaged over 18 trials) of the six patients with left-sided extinction (GVS group) in the Quality Extinction Test (QET) across the different experimental conditions for the left arm and in relation to lesion chronicity (months), separately for application of different tactile stimuli (A) and of identical tactile stimuli (B)**. Abbreviations: see legend of Figure 2. Mo, months. For patient codes and lesion chronicity, see Table 1.

Moreover, we explored to which extent the improvement of tactile extinction in the experimental group was related to chronicity of the lesions, as this differed widely in the six patients (from 2.3 to 71.2 months). Figure 4 shows the individual graphs for the left hand extinction errors, respectively for every patient and separately for different and identical trials. All patients showed a reduction in extinction errors for different as well as for identical stimuli, either in the L-GVS or in the R-GVS condition, independently of chronicity. We calculated Pearson correlations between the chronicity of lesions and the improvement in tactile extinction for both GVS polarity conditions as compared to averaged scores of the two Baselines (mean of extinction errors in Baseline 1 and Baseline 2), and did not find any significant coefficients (smallest *p* = 0.195, largest *r_p_* = 0.61).

In summary, patients in the GVS and control group did not differ in their right-sided extinction scores for different as well as for identical stimuli in and across any of the GVS conditions, respectively TP. By contrast, concerning left-sided extinction scores, only patients in the GVS group showed differences between experimental conditions, thus ruling out learning, test repetition, or other unspecific effects. When compared against averaged Baseline scores, L-GVS improved transiently the tactile identification of different (improvement of 50%) and of identical materials (47%) and also R-GVS led to a reduction of left-sided extinction rates for different (37%) and identical stimuli (55%). These effects remained stable at the follow-up test 2.8 months later (different: 37% over averaged Baseline scores; identical: 58% over averaged Baseline scores). Sham-GVS had no significant effect.

## Discussion

The present study showed the following results: (i) GVS significantly reduced tactile extinction, this effect being independent of the chronicity of lesions. (ii) We did not find polarity-specific effects of GVS on tactile extinction, as L-GVS and R-GVS significantly improved left-sided extinction to a similar extent. (iii) A small number of GVS sessions was sufficient to induce lasting changes in tactile extinction that remained stable for at least 2.8 months post-stimulation. (iv) Sham-GVS or retesting had no effect on tactile extinction, nor was there any reduction of GVS thresholds during the course of the study. (v) Patients showed differences in identification of different and identical stimuli, respectively before treatment as well as during GVS.

### Effects of GVS on bodily awareness

Both, L-GVS and R-GVS significantly reduced left-sided tactile extinction in the identification of different and identical tactile fabrics delivered during DSS. Improvements in left hand extinction during and after GVS did not occur at the expense of right-hand errors (which remained completely unchanged throughout the study). Initially, previous studies found an asymmetry of the cortical vestibular system (Dieterich et al., 2003). Therefore, galvanic inhibition of the L-GVS with excitation of the R-GVS results in *right* vestibular cortex activation whereas galvanic inhibition of the R-GVS with excitation of the L-GVS activates vestibular cortices *bilaterally*, at least in healthy subjects (Fink et al., 2003). Thus, L-GVS may lead to a more widespread cerebral activation in *both* hemispheres that could result in a greater effect on tactile extinction as compared to R-GVS. One explanation for the comparable efficiency of R-GVS and L-GVS in reducing left hand tactile extinction could be that even the weaker, unilateral activation induced by R-GVS was sufficient to improve left hand tactile extinction. In contrast, in more severe disorders such as left multimodal neglect, stronger activations may be necessary, so that R-GVS may induce less or even no significant beneficial effects (e.g., on deficits in left arm position sense, cf. Schmidt et al., 2013). Additionally, some theories view extinction as a mild form of neglect (Kaplan et al., 1995), which may be more easily influenced by any type of GVS, regardless of polarity.

In our six experimental patients we found stable improvements in tactile extinction by GVS for at least 2.8 months (Follow-up 1; improvement of 37% over averaged Baselines during different tactile stimulation; improvement of 58% over averaged Baselines during identical tactile stimulation). Furthermore, five out of these patients performed the QET in a second follow-up session 336 days [=11.2 months (mean); range: 90–750 days] after Follow-up 1. We found a persistent effect of GVS on tactile extinction performance even at this later time-point of measurement which confirms the enduring effect of this vestibular stimulation method. The persistence of improvement in tactile extinction after GVS at follow-up assessments could be explained by principles of synaptic plasticity, e.g., long-term potentiation (LTP), a well-known phenomenon of neuroplasticity induced by direct-current-stimulation (Utz et al., 2010) and make in a promising rehabilitation treatment.

Finally, Sham-GVS did not significantly influence tactile extinction, thereby ruling out placebo or unspecific effects of the stimulation procedure. Moreover, the observed modulating effects are unlikely to result from mere attentional cueing because the patients could neither feel the stimulation nor discriminate between different GVS conditions because of subliminal stimulation. This is confirmed by the fact that comparable retesting of extinction *without* GVS in the control group had no effect on extinction. Spontaneous recovery can also be ruled out as an explanation as there was no change in the QET across the two Baselines before treatment and such recovery should have occurred in both patient groups which was not found. The individual threshold was unchanged across stimulation sessions and patients did not report any adverse effects in every GVS sessions, indicating that we stimulated subliminally in each GVS session. This fact rules out potential attentional cueing effects induced by supra-threshold stimulation. Independently of this, future studies might consider whether repetitive GVS may reduce somatosensory thresholds, e.g., in pressure sensitivity, two-point discrimination, or other somatosensory capacities, as this was not the focus of the current study.

### Different vs. identical tactile stimuli

As stated in the description of the QET (see above) and shown by our data it is more difficult to identify (among six different materials) and name two *different* materials than two *identical*. In the latter condition the subject even may adopt an implicit (even unconscious) strategy where he/she decides that if both stimulations were “comparable” both materials must represent the same material. This strategy is not applicable during DSS with different tactile stimuli. We do not know whether such a mechanism was at work since all patients denied having used such a strategy during testing. Nevertheless, a closer look at the data shows a kind of double dissociation: *R-GVS* improved left-sided tactile extinction of *identical* stimuli to a greater extent (+55%) as compared to different stimuli (+37%), whereas *L-GVS* reduced left-sided extinction errors during stimulation with *different* stimuli to a greater extent (+50%) as compared to identical stimuli (+47%), although these differences between the groups and materials were not significant. This trend corresponds to the results in our previous case studies (Kerkhoff et al., 2011), though not to a significant extent. It seems plausible to assume that R-GVS is strong enough to modulate extinction of *identical* trials but only L-GVS leads to such a strong bi-hemispheric activation that it can influence extinction in the more demanding condition with *different* tactile materials in the QET. As discussed in our earlier case studies (Kerkhoff et al., 2011), the greater effect of L-GVS on different stimuli in the QET could be explained by the fact that L-GVS activates perisylvian cortices in *both* hemispheres, hence also in the language-related areas of the left perisylvian cortex of the patients that is needed for the verbal output during extinction testing. In line with this hypothesis, the developers of the QET (Schwartz et al., 1979) proposed that “During the extinction tests a response mechanism in the left (speech) hemisphere bases its perceptual output on the relative strengths of two simultaneous sensory inputs. Damage at any point in the channel from the periphery to the response mechanism weakens one signal in comparison to the other, resulting in a response bias favoring the stronger stimulus” (Schwartz et al., 1979, p. 681f). Thus, GVS may have modulated the different “strengths” of the unimanual tactile inputs during extinction testing at various processing stages in the brain.

### Implications for rehabilitation

Apart from the above discussed mechanisms of GVS on tactile extinction, GVS may speed up tactile discrimination learning *during* DSS, which did not occur after mere test repetitions *without* GVS, as shown in Figures 2 and 3 in the control group. This may reflect another interesting and testable hypothesis for future studies as somatosensory deficits and extinction are frequently encountered after brain damage (Van Stralen et al., 2011). Due to long-lasting effects of GVS, it may be used as an add-on-treatment in combination with other trainings of somatosensory deficits for rehabilitation. Whatever the precise mechanism of improvement induced by GVS, our results are compatible with the hypothesis that GVS permanently changed the relative strengths of the tactile inputs from both hands. This may result either from an enhancement of left hand-input and/or a reduction of right-hand-input, or another kind of re-weighting of both inputs. Importantly, the improved discriminations observed on the left hand did not occur at the expense of a deterioration in right-hand performance. Moreover, as GVS had similar beneficial effects on left-sided tactile extinction in all of our six patients (see Figure 4) – despite their different brain lesions and their different lesion chronicity (see Table 1) – it appears that treatment effects induced by GVS do not rely on a particular lesion area in order to occur. This makes GVS an interesting candidate for further treatment studies of tactile extinction and related body cognition disorders.

Our study extends earlier findings on the modulation of tactile extinction using the same extinction test but another stimulation technique: RPMS (Heldmann et al., 2000). Following one session of RPMS, left hand tactile extinction was on average reduced by some 28% in seven extinction patients while right-hand scores remained unchanged. In contrast, attentional cueing to the left side in a comparable group with seven other extinction patients had no beneficial effect on left hand extinction scores but increased right-hand errors significantly. Due to clinical limitations no repetitive RPMS sessions could be delivered in these patients so that the authors could not evaluate longer-lasting therapeutic effects of RPMS. As this technique is widely available in many neurology or neurorehabilitation clinics (which is, in fact, technically identical to transcranial magnetic stimulation, TMS), RPMS, and GVS may induce similar therapeutic effects on tactile extinction. Interestingly, both activate – among other brain areas – motor cortex and parietal areas (Struppler et al., 2007; Lopez et al., 2012a), the latter being one cortical projection area of somatosensory pathways and hypoactivation of SII is associated with tactile extinction (Remy et al., 1999). Both RPMS and GVS might thus alleviate tactile extinction – transiently or permanently – by increased activation of this under-activated brain area. This mechanism may occur either by an improved “bottom-up interpretation” of tactile information from both stimulated hands in extinction, or by improved “top-down interpretation” of these signals, or by both mechanisms simultaneously, as suggested recently by Ferrè et al. (2011a). Principles of synaptic plasticity, e.g., LTP, induced by repetitive stimulation may then lead to lasting changes, both on the physiological and behavioral level.

### Vestibular cortex and vestibular stimulation

Neurophysiological studies in primates all have indicated the parietal lobe as the main projection area of vestibular input, with other additional subcortical and cortical projection zones (for a review, see Lopez et al., 2012b). Electrical stimulation of the vestibular nerve showed a cortical projection to Brodman area 2 (Schwarz and Fredrickson, 1971) and evoked potentials showed cortical activations in Brodman area 3 (Ödkvist et al., 1974). Functional imaging studies using caloric vestibular stimulation show activations in areas of the perisylvian cortex including the insula and retroinsular cortex, the temporo-parietal cortex, the putamen, somatosensory area II (Bottini et al., 2001), as well as in the intraparietal cortex (Suzuki et al., 2001; Chokron et al., 2007). In accordance with these activations, numerous studies using caloric vestibular stimulation have shown a beneficial influence on neglect and neglect-related disorders such as tactile extinction (Vallar et al., 1993), somatoparaphrenia (Rode et al., 1992), or unawareness of hemiplegia (for a review, see Vallar et al., 2003). Interestingly, caloric vestibular stimulation modifies the body schema (tactile distance estimation and hand-shape judgments; Lopez et al., 2012b) and also enhances somatosensory functions transiently in the *healthy* brain, when very demanding, fine discriminations (detecting a stimulation with a von Frey hair) were required (Ferrè et al., 2011a,b). The authors speculated that vestibular stimulation might have achieved this increase in sensitivity by way of a cross-modal enhancing mechanism. Such mechanisms are well-known for other modalities, e.g., visual and auditory integration (Meredith and Stein, 1986).

## Conclusion

In conclusion, two sessions of real (verum), subliminal GVS induced a significant and enduring improvement in tactile extinction in six patients with right-hemisphere brain lesions, thus enhancing tactile awareness permanently on their contralesional body side. This beneficial effect ranged up to a level of postsensory processing of bilateral tactile input onto a verbal output level. To our knowledge, this is the first study that reports a long-lasting, therapeutic reduction of tactile extinction in a patient group following a systematic intervention. As subliminal GVS produced no serious side effects in this and other studies (Utz et al., 2011b) it is convenient for *repetitive* stimulations, i.e., in treatment studies. Moreover, subliminal GVS is painless, non-invasive, safe, easily applicable, and elegantly allows the realization of placebo/Sham stimulation without the patient being aware of any stimulation or of the cessation of stimulation. Furthermore, GVS shows other beneficial modulation effects in treatment of neglect, extinction, and related disorders: it reduces, albeit transiently, the ipsilesional bias in line bisection (Utz et al., 2011a), normalizes deficits in left arm position sense in left neglect within one 20-min sessions of GVS for at least 20 min post-stimulation (Schmidt et al., 2013), and multi-session GVS reduces tactile related spatial deficits in a case study of a pusher patient with left neglect (Volkening and Keller, 2012) as well as deficits in target cancelation in two patients with visuo-spatial neglect (Zubko et al., 2013). Therefore, repetitive GVS is a promising treatment approach that could enhance the rehabilitation of body- and space-related disturbances associated with right-hemisphere lesions.

## Conflict of Interest Statement

The authors declare that the research was conducted in the absence of any commercial or financial relationships that could be construed as a potential conflict of interest.

## Abbreviations

DSS, double simultaneous stimulation; GVS, galvanic vestibular stimulation; L-GVS, left-cathodal/right-anodal GVS; mA, milliAmpere; R-GVS, right-cathodal/left-anodal GVS; TP, time-point of measurement; QET, quality extinction test.
